# Nanoparticles as potential new generation broad spectrum antimicrobial agents

**DOI:** 10.1186/s40199-015-0125-6

**Published:** 2015-09-02

**Authors:** Clarence S. Yah, Geoffrey S. Simate

**Affiliations:** Department of Biochemistry and Microbiology, Nelson Mandela Metropolitan University, Port Elizabeth, South Africa; Department of Epidemiology, Johns Hopkins Bloomberg School of Public Health, E7146, 615 N. Wolfe Street, Baltimore, 21205, MD USA; School of Chemical and Metallurgical Engineering, University of the Witwatersrand, P/Bag 3, Wits 2050, Johannesburg, South Africa

## Abstract

The rapid emergence of antimicrobial resistant strains to conventional antimicrobial agents has complicated and prolonged infection treatment and increased mortality risk globally. Furthermore, some of the conventional antimicrobial agents are unable to cross certain cell membranes thus, restricting treatment of intracellular pathogens. Therefore, the disease-causing-organisms tend to persist in these cells. However, the emergence of nanoparticle (NP) technology has come with the promising broad spectrum NP-antimicrobial agents due to their vast physiochemical and functionalization properties. In fact, NP-antimicrobial agents are able to unlock the restrictions experienced by conventional antimicrobial agents. This review discusses the status quo of NP-antimicrobial agents as potent broad spectrum antimicrobial agents, sterilization and wound healing agents, and sustained inhibitors of intracellular pathogens. Indeed, the perspective of developing potent NP-antimicrobial agents that carry multiple-functionality will revolutionize clinical medicine and play a significant role in alleviating disease burden.

## Introduction

In recent past, microbial infections have become a global health burden due to emerging and resistant strains of viruses [1], bacteria [2], pathogenic fungi [3] and protozoa [4] defying clinical treatment. Consequently, this has culminated into prolonged treatment, higher health expenditure, mortality risk, and low life expectancy [2]. In view of ineffective antimicrobial agents, there is need to seek new alternative and safer antimicrobial agents against these “super bugs” of viruses, bacteria, fungi and protozoa. With the development of biomedical nanomaterials, new antimicrobial agents have begun to emerge either as novel and/or augmenting the activities of the current conventional antimicrobials. This is motivated by the vast physiochemical and functionalization (ligand attachment) properties of nanoparticles (NPs) [5–7]. The NPs physiochemical properties are highly diverse in nature and are highly applicable in biomedical field including antimicrobial and drug delivery [6, 8, 9]. Some examples of these biomedical NPs include silver nanoparticles (AgNPs) [10], carbon nanotubes (CNTs) [11], gold NPs (AuNPs) [12], zinc oxide NPs (ZnO-NPs) [13], and iron oxide NPs (FeO-NPs) [14].

**Fig. 1:**
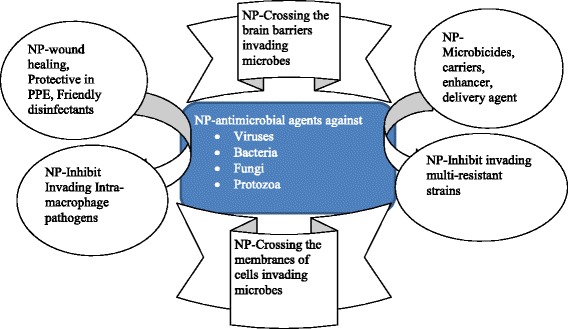
Multiple functionality and broad spectrum activities of nanoparticles antimicrobial agents. The diverse vast antimicrobial uses of nanoparticle bioconjugates. Used for wound healing, use as anticancers, anti multi-resistant pathogens, aid drugs to cross the blood brain barriers, help in the inhibition of microbes that hide in macrophages. NP = acronyms for Nanoparticles. PPE = Personal Protective equipment incorporated with nanoparticles capable of destroying microbes

The antimicrobial actions of NPs include cidal destruction of cell membranes, blockage of enzyme pathways, alterations of microbial cell wall, and nucleic materials pathway [1]. However, the antimicrobial mechanisms of the actions are yet to be fully elucidated since some of the NPs drugs are still at their infancy. The high potency of NPs antiviral, antibacterial, antifungal and antiprotozoal activities may revolutionize and bring another turning point in pharmacological therapy. In that regard, this review looks at the status quo of nanomaterials as alternative antimicrobial agents in terms of their broad spectrum ability, the crossing of difficult membrane barriers, delivery and sustained inhibition of intracellular pathogens and sterilization abilities as shown in Fig. 1. This perspective status quo of NP antimicrobial agents with multiple functions will play a significant impact on the treatment of diseases.

## Broad spectrum nanoparticle-antimicrobial agents

**Fig. 2:**
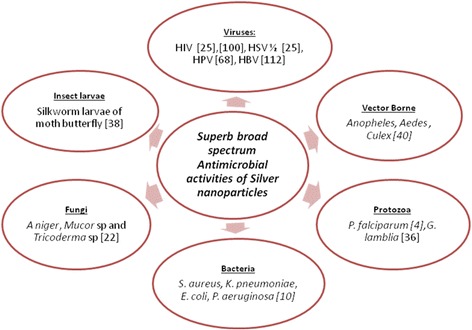
Broad spectrum NP-antimicrobial activities of silver nanoparticles. The Figure describes the antimicrobial spectrum of silver bio-conjugate nanoparticles against diverse genera of microorganisms. HIV = Human immunodeficiency virus, HSV = Herpes Simplex Virus 1, HPV = Human papillomavirus, HBV = Hepatitis B virus, *P. falciparum = Plasmodium falciparum, G. lamblia = Gardia lamblia, S. aureus = Staphylococcus aureus, E. coli = Escherichia coli, P. aeruginosa = Pseudomonas aeruginosa,* sp = species

The global emergence of multidrug-resistant microorganisms (viruses, bacteria, fungi and protozoa) has made conventional treatment of infectious diseases difficult. Therefore, the discovery of alternative new classes of antiviral [15], antibiotics [16], antifungal [17], and antiprotozoal [18] agents that can treat resistant strains is paramount. Research has shown that these emerging broad-spectrum antimicrobial nanomaterial can knock-out diverse pathogenic organisms of different phyla, across diverse and/or within species of viruses, bacteria and fungi [19–22]. For example, Fig. 2 shows the broad spectrum NP-antimicrobial effect of AgNPs. The AgNP antimicrobial agent has multi-functionality of antibacterial [22], antifungal [22], antiviral [23], anti-parasitic [4], and anti-inflammatory properties [14, 24].

One of the mechanisms of NP-antimicrobial actions is cell wall lysis. For example, a study by Addae et al. [12] in an attempt to produce a transducer agent for photothermal therapy (PTT) found the destruction of *Bacillus* species cell membranes when treated with Au/CuS NPs. The destruction of *Bacillus* species in this study proved that Au/CuS NPs are potent NP-antimicrobial agents.

The NPs are potential broad spectrum antibiotics because they can inhibit wide range of multidrug-resistant strains of bacteria that have defied most antibiotic treatment. For example, in the study by Adeli et al. [10] it was found that AgNPs were able to inhibit pan-multidrug resistant strains of *S. aureus, K. pneumoniae*, *E. coli*, and *P. aeruginosa* that were resistant to all the antibiotic drugs including imipenem. Another similar study by Kathiravan et al. [22] showed that AgNPs can inhibit both bacteria (*S. aureus, E coli, B subtilis*) and fungi species (*A niger*, *Mucor* sp and *Tricoderma* sp). In addition, earlier findings by Fayaz et al. [25] showed that the AgNPs-coated condom have antiviral (against HIV-1 and HSV-1/2), antibacterial (against *E. coli*, *S. aureus*, *M. luteus*, *K. pneumonia*) and anti-fungi (against *Candida* spp.) properties. This suggests that AgNPs can be used to treat all multi-drug resistant pathogens from diverse phyla from all clinical sources.

The broad spectrum antimicrobial activities have also been demonstrated by CNTs. For example, a study by Tank et al. [26] showed that silica coated silicon nanotubes (SCSNTs) exhibit enhanced antimicrobial activities when compared to other non-silica coated silicon nano-particles. Other studies also found that CNTs containing lysine such as multiwalled CNT (MWCNT)-epilsonpolylysine [27], and SWCNT-poly(L-lysine) (PLL), and poly(L-glutamic acid) [28] exhibit very strong broad antimicrobial activities against a wide range of bacteria. A study by Amiri et al. [29] showed MWCNT-lysine exhibiting very strong broad antimicrobial activity against *S. aureus*, *S. agalactiae, S. dysgalactiae, E.coli, K. pneumonia* and *Salmonella typhimurium.*

In addition to antimicrobial activities, hybrids of nanomaterials such as cholesterol-containing liposomes phytonanosilver and CNTs have been found to exhibit high antioxidant activity as well as antimicrobial activities against *E. coli, Staphylococcus aureus* and *Enterococcus faecalis* [30]. This shows that when two or more NPs are combined, they tend to enhance the broad spectrum activity of the nano-antimicrobial agents. The hydrid behaviour was equally found when CNTs and AgNP-based nanomaterials were combined and the resulting hybrid biocomposite was found to exhibit stronger and excellent antimicrobial properties [27]. Similarly, chitosan-CNT hybrid showed excellent antimicrobial activities against bacteria and fungi [9]. Other CNTs antimicrobial hybrids include ZnO coated MWCNTs (ZnO/MWCNTs) [13], Triad CNT-NPs/Polymer nanocomposites [11], functionalized MWCNTs-CdS and functionalized-MWCNTs-Ag2S [31], and CdTe QDs/single-walled aluminosilicate nanotubes [32]. Furthermore, Cefalexin-immobilized multi-walled CNTs have been found to broadly enhance the antimicrobial activities against a wide range of pathogens including *E. coli, P. aeruginosa, S. aureus* and *Bacillus subtilis* [8] as shown in Table 1. The combination of AgNPs and CNTs including MWCNT-AgNPs [33] on fiber membrane has also been found to enhance the filtration and antimicrobial potentials against all types of bacteria. In addition, Poly(N-vinylcarbazole) (PVK)-SWCNT nanocomposite coated membrane for water purification were found to destroy all bacterial species including spore forming organisms such as *Bacillus subtilis* [34]. Apart from filtration and demonstration of antimicrobial activities the MWCNT-AgNPs hybrid composite membrane has been found to significantly reduce biofilm formation which can easily be extended to other types of support membranes [33]. Table 1 summaries the types of NPs and their susceptibility to various organisms.

**Table 1:** Summary of the types of nanoparticles susceptibility to organisms

Type of NP	Method of NPs characterization	Size of NP	Types organisms inhibited	Outcome	Toxicity	Author
Fe-Oxide NP & AgNP	UV–vis spectroscopy, Fourier Transform Infrared Spectroscopy (FTIR), Transmission Electron Microscopy (TEM)	Fe-oxide NP 20–40 nm, AgNP 10–20 nm	*Bacillus, E. coli* and *Staphylococcus* species	Fe-Oxide NPs were sensitive against *Bacillus, E. coli* and *Staphylococcus* species*.*	The very smaller size AgNP were toxic against the pathogens	[14]
Ag NPs.	TEM, Field Emission Transmission Electron Microscopy (FESEM), FTIR, UV–Vis spectra, Raman spectroscopy, X-ray Difraction (XRD)	Average 18–20 nm	*Escherichia coli, Pseudomonas* spp. *Bacillus* species, *Staphylococcus* species, *Aspergillus niger, Aspergillus flavus*, *Penicillium*	Inhibited the growth and multiplication of *E. coli, Pseudomonas* species, *Bacillus* spp. and *Staphylococcus* species, *A. niger, A. flavus, Penicillium* spp	ND	[98]
Silver, chitosan, and curcumin nanoparticles	NA	-	*Giardia lamblia*	The highest effect was achieved by combining the three nanoforms. The parasite was found to be eradicated from stool and intestine.	None of the nanoparticle exhibited toxic effect	[36]
AgNPs	UV spectra, TEM	2−30 nm; averagely 20 nm	*S. aureus, Klebsiella pneumoniae*, *Escherichia coli*, and *Pseudomonas aeruginosa*	The AgNPs produced had strong antibacterial effect against all the pathogenic bacteria	ND	[10]
polyvinylpyrrolidone (PVP)-coated silver nanoparticles	-	1–10 nm	HIV-1	PVP-coated AgNP exhibit potent cyto-protective and post-infected anti-HIV-1 activities toward Hut/CCR5 cells.	ND	[99]
PVP-coated silver nanoparticles	-	30−50 nm	HIV-1	PVP-coated AgNPs Inhibited cell-associated HIV-1 and cell-free HIV-1 transmission.	PVP-coated AgNPs were non toxic to cells explant	[100]
mercaptoethane sulfonate (MES)-coated silver and gold nanoparticles	-	4 nm	Herpes simplex virus type 1 (HSV-1)	The MES-coated silver and gold nanoparticles inhibited HSV-1 infection in cell culture	The MES-coated silver and gold were non toxic to host cells	[101]
PVP-coated silver nanoparticles	-	69 nm +/− 3 nm	Respiratory syncytial virus (RSV)	Inhibited RSV infection	showed low toxicity to cells	[102]
AgNP and polysaccharide-coated AgNP	-	10−80 nm	Monkey pox virus (MPV)	The AgNPs of approximately 10 nm inhibit MPV infection in vitro, as an anti-viral	Non of te GgNPs were cytotoxic (Vero cell monolayer sloughing)	[103]
AgNPs	-	10−50 nm	Hepatitis B virus (HBV)	AgNPs inhibited in vitro HBV RNA and extracellular virions	ND	[104]
AgNPs and polysaccharide-coated AgNP	-	10 nm	Tacaribe virus (TCRV)	AgNPs inhibited the TCRV infection in vitro	ND	[105]
Ag-NPs-coated PUC	High resolution Scanning Electron Microscopy (HrSEM), UV Spectra	30–60 nm	*E. coli*, *S. aureus*, *M. luteus*, *K. pneumoniae*, and *Candida tropicalis*, *Candida krusei*, *Candida glabrata*, and *Candida albicans and* HIV-1	Ag-NPs-coated PUC with HIV-1 and HSV-1/2 was able to inactivate their infectiousness as well as bacterial and fungal species	ND	[25]
Mycosynthesized silver nanoparticles	UV spectra, TEM, Nanosight-LM 20;	4−46 nm	HSV 1 and 2 and with human parainfluenza virus type 3.	Smaller-sized AgNPs were able to inhibit the infectivity of the viruses	ND	[23]
AgNPs	UV–vis spectroscopy, SEM, TEM, FTIR and XRD.	18 to 45 nm with an average size of 32 nm	*Anopheles stephensi, Aedes aegypti*, and *Culex quinquefasciatus*	AgNPs showed biolarvicidal effect to *A. stephensi, A. aegypti,* and *C. quinquefasciatus.*	ND	[39]
AgNPs	UV–vis spectroscopy, SEM, FTIR and XRD.	41–60 nm.	*Anopheles stephensi, Aedes aegypti*, and *Culex quinquefasciatus*	The AgNPs were effective in destroying the vectors of mosquito vector blood born parasites	ND	[40]
AgNPs	Atomic force microscopy (AFM), UV–vis spectroscopy, FTIR	60–95 nm	3 instar larvae of Culex quinquefasciatus	AgNPs exhibited high mortality against larvae of *Culex quinquefasciatus*	ND	[78]
AgNPs	UV–vis spectroscopy, SEM, energy-dispersive X-ray (EDX) spectroscopy.	43.52 to 142.97 nm	Aedes aegypti	The Bt-AgNPs showed larvicide effect against mosquito larva A. aegypti	ND	[106]
Polyvinyl-N-carbazole (PVK) and single-walled carbon nanotubes (SWNTs) (PVK:SWNT)	UV vis spectra, FTIR, SEM	NA	*E. coli* MG 1655 and *B. subtilis-*102	The nano-composite showed antimicrobial activity against both Gram-positive and negative bacterial isolates.	The PVK-SWNT were non toxic to fibroblast cells	[34]
MWCNT-lysine functionalized	FTIR, Thermal gravimetric analysis (TGA), Raman spectra and TEM	N/A	*S. aureus, Streptococcus agalactiae, S. dysgalactiae, E. coli, K. pneumonia, S. typhimurium*	The functionalized MWCNT with lysine expressed high antimicrobial effect against all bacterial cells	ND	[29]
MWCNT-AgNPs	Inductively coupled plasma atomic emission spectroscopy (ICP-AES), XRD, FTIR	3 to 30 nm	*Escherichia coli*	MWCNT-AgNPs exhibited strong antimicrobial activities and reduce biofilm formation.	ND	[33]
Silicon nanotubes (SNTs), silicon nanoparticles (SNPs)	SEM–EDX, TEM, Brunauer-Emmett-Teller (BET), STM, Raman spectroscopy.	average diameter of 14	Multidrug-resistant *Staphylococcus aureus*	SCSNTs were effective in limiting the growth of multidrug-resistant *S aureus*	ND	[26]
Ag–Fe/SWCNTs	TEM, SEM, XRD, Raman spectra	1−10 nm Ag-Fe NP dispersed and tightly attached to the outer surfaces of SWCNTs	*Escherichia coli.*	Purified Ag–Fe/SWCNT hybrid nanoparticles were effective against *E. coli.*	ND	[104]
SWCNTs combine with H_2_O_2_ or NaOCl	TEM, SEM-EDX	SWCNTs 1–1.5 nm	*Bacillus anthracis* Spores	The combined effect of SWCNTs and H_2_O_2_ or NaOCl exhibited sporicidal effect on *B. anthracis* spores	ND	[87]
SWNT/PLL/PGA	Uv spectra, TEM, SEM, Quartz crystal microgravimetry	SWNT is 0.8–1.2 nm	*E. coli* and *S. epidermidis*	SWNT/PLL/PGA highly inactivated *E. coli* and *S. epidermidi*s	ND	[28]
Zirconia (ZrO2) nanoparticles	SEM, EDX, AFM, U vis spectra, FTIR	50e100 nm, average size 50 nm	*Staphylococcus aureus, Escherichia coli, Candida albicans, Aspergillus niger*	Zirconia (ZrO2) nanoparticles exhibited antifungal and antibacterial against the test organisms.	ND	[111]
Au/CuS core/shell nanoparticles (NPs)	HRTEM, SEM, energy dispersive X-ray spectroscopy (EDS)	2–5 nm.	*B. anthracis* spores and cells	The Au/CuS NPs were highly efficient in inactivating *B. anthracis* cells, but not effective to the spores.	ND	[12]
Sialic-acid functionalized gold nanoparticles	TEM	2 nm and 14 nm	Influenza virus	The NPs inhibition influenza virus infection	The functionalized AuNPs were nontoxic to the cells	[107]
Titanium dioxide nanoparticles (TiO2 NPs)	XRD, FTIR, SEM, EDX, AFM.	Average size of 70 nm.	Pediculus humanus capitis De Geer (Phthiraptera: Pediculidae); larvae of cattle tick Hyalomma anatolicum (a.) anatolicum Koch (Acari: Ixodidae), and fourth instar larvae of malaria vector Anopheles subpictus Grassi (Diptera: Culicidae).	The TiO_2_ NPs showed significant mortality against the vectors borne organisms	ND	
Chrysosporium tropicum mediated silver and gold nanoparticles	Microscan reader, XRD, TEM, SEM	AuNPs: 2–15 nm and AgNP: 20–50 nm	*Aedes aegypti* larvae.	The AuNPs used as an efficacy enhancer shown mortality 3 times higher *Aedes aegypti* larvae.	ND	[41]
Zinc oxide nanoparticles (ZnO NPs)	UV–visible spectroscopy, XRD, FTIR, SEM	60–120 nm.	larvae of cattle tick Rhipicephalus (Boophilus) microplus, Canestrini (Acari: Ixodidae); head louse Pediculus humanus capitis, De Geer (Phthiraptera: Pediculidae); larvae of malaria vector, Anopheles subpictus, Grassi; and filariasis vector, Culex quinquefasciatus, Say (Diptera: Culicidae). R. microplus larvae	The ZnO NPs had significant inhibitory effect on the parasites	ND	[110]
Cobalt nanoparticles (CoNPs)	XRD, FTIR FESEM with energy dispersive X-ray spectroscopy, and TEM	average size of 84.81 nm.	malaria vector Anopheles subpictus and dengue vector *Aedes aegypti (Diptera: Culicidae).*	The larvicidal effect was observed in the cobalt acetate solution and against the *A. subpictus* and *A. aegypti*	ND	[108]
Copper(II) nanohybrid solids, LCu(CH_3_COO)_2_ and LCuCl_2_	TEM, dynamic light scattering, and IR spectroscopy	5–10 and 60–70 nm of LCu(CH_3_COO)_2_ and LCuCl_2_	*Plasmodium falciparum* (MRC 2).	The two compounds showed significant antimalarial activities against the parasites	The copper(II) nanohybrid solids were nontoxic to human hepatocellular carcinoma cells	[109]

### Nanoparticle anti-parasitic effect

Despite the efforts made in the treatment of parasitic infections, infections by parasites particularly those of giardiasis, schistosomiasis, trypanosomiasis, malaria, leishmaniasis, dengue fevers, Japanese encephalitis, and filariasis continue to increase particularly in tropical and low income countries [24, 35, 36]. The problems associated with parasitic infections include drug toxicity, ineffectiveness, and developments of resistance to conventional anti-parasitic drugs. Furthermore, treatment costs are high, thus limiting supply of drugs in low income countries [37]. As a results of the limitation in anti-parasitic drugs, newer approaches such as nano-biotechnology have shown significant improvement in the treatment of parasitic infections [24]. This is based on the unique properties of NPs including those of AgNPs, AuNPs, chitosan, selenium oxide, and other metallic oxide based NPs that have shown excellent inhibitory effects against parasitic infections including insect larvae [24, 35–38]. 

Parasites such as *Leishmania* can reside and survive inside macrophages without being exposed to cell damage by reactive oxygen species (ROS) and anti-parasitic drugs [37]. However, AgNPs, because of their trans-membrane mechanisms and sustained anti-parasitic delivery, can inhibit intracellular *Leishmania* and enhance their destruction via ROS [37].

Other NPs including the combination of silver, chitosan, and curcumin nanoparticles have been used in the treatment of *Giardia lamblia* as demonstrated in experimental animals [36]. The findings also showed that *Giardia lamblia* can be successfully eradicated from stool and intestine [36]. The potential of NPs if fully optimized may lead to the development of newer synergic antimicrobials where two or more nano-antimicrobials are combined to generate an effective efficacy in the eradication and probably the elimination of parasitic infections. Some studies have shown that modified *Plasmodium berghei* sporozoite (Tg-Pb/PfCSP) and self-assembling protein NP (SAPN) vaccine presenting *Plasmodium falciparum* circumsporozoite protein epitopes (PfCSP-SAPN) can stimulate humoral and cellular responses against *Plasmodium falciparum* using the complement classical pathway cascade [4]. The results indicates the potential application of the circumsporozoite protein epitopes (PfCSP-SAPN) in the development of protective effector memory CD8+ T-cells [4] capable of generating strong long-lived IgG.

### Nanoparticle anti-vector borne diseases

As a result of the increase in the prevalence of vector borne diseases, the production of environmentally friendly and safe NP insecticides synthesized from plants are currently available. These include those of AgNPs synthesized from the leaf extracts of *Heliotropium indicum* [39], and *Azadirachta indica* [40]. These insecticides have shown maximum efficacy against blood feeding mosquitoes of *Anopheles stephensi, Aedes aegypti*, and *Culex quinquefasciatus* [39, 40]. This shows that eco-friendly NPs have the potential of controlling vector transmitted infections that have significantly contributed to disease burden, social debility, poverty and death in mostly low income countries [39, 40]. However, due to NPs non-specific actions to environmental organisms, this may deter their usefulness as vector control agents [40–42].

## Wound healing and nanoparticles

Wound dressing and wound healing are very important components of reducing morbidity and mortality of wound related burden. A wound is a debilitated tissue that results from a breakdown in the skin giving rise to a physiological condition for microbial manifestation including opportunistic pathogens [43, 44] affecting wound healing [45]. Depending on the degree of wound, whether acute or chronic, wound care is necessary to reduce infection or abnormal bacterial presence that may cause stress and other health consequences [44, 46]. Over the years, wound dressing and healing have been problematic to clinicians [46]. Because there is no single appropriate wound dressing material that can act as a potent sterile antimicrobial agent capable of absorbing excess exudate, preserving the wound from external sources of infection, preventing excess heat at the wound, impermeable to gases, and a dressing that is easy to remove without further trauma to the wound [47] has complicated wound healing. Wound dressing materials such as gauze are associated with painful removal and may cause trauma and associated stress [48].

Nevertheless, the research on NPs in wound dressing materials has come at an opportune time. The NP wound dressing materials provide biocompatible antimicrobial agents that are inexpensive, soft, and flexible, and conform to the contours of the body [49, 50]. For example, AgNPs wound dressing antimicrobial nanomaterials have been introduced to supplement traditional wound dressing because the slow release of the AgNPs allow the dressing to be changed less frequently, but is highly effective and efficient in wound healing with less antimicrobial resistance [49]. Furthermore, a study by Guidelli et al. [51] showed that natural latex rubber blended with AgNPs gradually released the AgNPs, but was useful in promoting and facilitating wound healing as well as the reduction in scar formation [49]. The AgNPs may also mediate wound healing via reduced mitochondria activity that does not affect the host cell viability with rapid re-establishment of the body integrity [52]. According to a study by Tian et al. [53] AgNPs exert positive broad spectrum antimicrobial properties by reducing wound inflammation, and modulation of fibrogenic cytokines.

Similarly, other findings by John and Moro [54] showed that NPs hydrogel wound dressing consist of methacrylate backbone and terminal hydroxyl group capable of providing versatile and excellent wound healing. This is because the NPs hydrogel dressing powders have thermal insulators capable of absorbing some of the blood or wound exudate, thus providing an impermeable potent antimicrobial environment to wound pathogens as well as protecting the wound from external contamination [50, 54]. The NPs hydrogel are cost effective, user friendly, easy to apply, do not adhere to the wound and have minimal need for secondary dressing [54].

Apart from AgNPs, other NPs equally used in wound healings include those of gold [55], curcumin-encapsulated NPs [56], chitin/nanosilver composite with good blood clotting ability [57], conjugated iron oxide NPs [58], and nitric oxide releasing NPs [59]. However, the significant acceleration of wound healing by nanomaterials still remains a mystery and the mechanisms of action are still to be fully elucidated and unfold.

## Nanoparticles microbicides activities

With the increase in sexually transmitted infections (STI) fuelling the HIV burden and other health problems, microbicides may be considered as alternative preventive methods of STI and HIV [60, 61]. Microbicides are antimicrobial agents that are self-applied on the vagina or rectum to protect against STIs [19, 62, 63]. Hence they act as chemical, biological and/or physical barriers that prevent transmission of pathogens during sexual intercourse [62, 64, 65]. They may be in gel, creams, rings, or films form and can be used with condoms, thus offering additional protection or used alone especially by those who do not appreciate the use of condoms [19]. The microbicides may be used by both HIV positive and heathy individuals to prevent transmission of the virus. Studies have shown that microbicides may provide prevention against HIV and STI infections for those practicing receptive anal and/or vaginal intercourse [63]. In addition, microbicides can provide individuals with protection especially those who are unaware of their partner HIV status including those on antiretroviral therapies (ART) and undetectable HIV viral load [63].

Research studies have shown that NPs-microbicides including those of dendrimer-nanoscale-microbicides hold potential safety efficacy against viruses [19, 66–68]. For example, the VivaGel™ (SPL7013Gel) dendrimer is carefully formulated against HIV and HSV and does not interfere with vaginal or rectal physiological pH [19, 69]. The dendrimer VivaGel™ microbicide is meant to disrupt and block viral attachment and/or prevent the viral adsorption from targeting cells of the rectum or vagina. In the case of HIV the gp120 of the virus are blocked from attaching to the CD4 receptors of human white blood cells [19]. In a study by Chonco et al. [60], it was found that carbosilane dendrimer microbicide are capable of exhibiting HIV thus blocking potential in epithelial monolayer in vitro model cells. Other dendrimers such as heparan sulfate-binding peptide were found to inhibit human papillomaviruses [68] thus, acting as promising antiviral microbicides.

## Nanoparticles inhibition of intra-macrophage pathogens

Pathogenic organisms that traverse cell membranes or reside in nerve cells cause persistence infections and, thus are difficult to treat [70]. Bacteria such as *Brucella, Mycobacterium, Listeria* species and viruses including HIV, and herpes simplex are intracellular pathogens that invade treatment and persistently exhibit latent infections [70–72]. Therefore, some drugs find it difficult to reach such cells, thus complicating the elimination and eradication of such microbial pathogens [73]. Some of the pathogens may invade cells and exist as intra-macrophage pathogens and central nervous infections escaping drugs action as well as immunological responses [71, 73]. Health care workers (HCWs) find it very difficult and frustrating when providing treatment to such intravascular disease causing pathogens due to failure of conventional antimicrobial drugs to destroy such organisms. Drugs for treating such diseases including HIV, encephalopathy and cerebrovascular infections may not lack potency, but due to shortcomings of poor or inefficient intracellular penetration and sustained drugs concentration, may limit treatment efficiency and efficacy [73]. The problems associated with such drugs may include lack of solubility and bio-distribution to reach target areas, thus do not have sufficient drug delivery profile.

Nano-drugs such as polymeric NPs, dendrimers, polymer micelles, and solid lipid NPs have been shown to exhibit excellent antimicrobial profiles and have potent ligand conjugates that improve the pharmacological and therapeutic profile of such drugs to cross such cell membranes, internalize and render efficient antimicrobial potentials [70, 71, 73]. The delivery process provide NP-drugs with multiple functions of carrier, delivery, and antimicrobial capabilities [71, 73, 74]. These attributes are due to the small size (1–100 nm), vast NPs-functionalization ability, and the robust physiochemical properties, even if biodegradability and the toxicological challenges may be hindering beneficial health outcomes [75, 76].

As mentioned earlier, organisms such *Brucella* species, *Mycobacterium tuberculosis* exist as intra-macrophage pathogen rendering standard treatment very difficult [71, 72]. For example, *Brucella* species usually invade, reside and survive within phagocytic, dendritic and trophoblast cells, thus making treatment potential very difficult to clinicians [71]. Similarly, *Mycobacterium tuberculosis* bacteria responsible for tuberculosis reside inside macrophage resulting into persistent tuberculosis [77]. The same effect has been demonstrated by herpes simplex virus that hides and resides in nerve cells causing latent herpes zosters infections [78]. The use of NPs could be beneficial for such treatments because of the NPs antimicrobials potentials, ease membrane crossing ability and delivery potentials of materials into such cells. They play the role of carrier, delivery and sustain antimicrobials effect in such cells. For example, AgNPs have huge biocidal effect and have been shown to cross the macrophage cell wall and inhibit intra-macrophage *Bacillus abortus*; a maternal bacterium that tend to resist treatment and causes perinatal morbidity during pregnancy [79].

Furthermore, some pathogens are highly resistant to extreme temperatures and difficult to be eliminated by antibiotics or other chemicals. Nanomaterials and other emerging materials have been reported to be potent antimicrobial agents capable of destroying such pathogens that are tolerant to extreme temperatures and resistant to treat with conventional antibiotics [80]. For example, SWCNTs coupled within 20 minutes near infrared (NIR) treatment significantly increases the potential effect of antimicrobials against *Bacillus anthracis* spores when compared to non NIR treated SWCNTs [67]. In addition, a study by Martínez-Gutierrez et al. [81] found that 24 nm AgNPs were not only potent antibacterial agents against resistant strains of bacteria, but also had anti-coagulation activities as well as inflammatory response in macrophages. This indicates that nanomaterials can easily be modified as efficient intravascular agent for the destruction of intravascular pathogens as well as delivery agents since they are capable of crossing membrane cell walls without any cell damage or harm [82]. However, the mechanisms of cell membrane or pathways used by the NPs antimicrobial agents in crossing/cell uptake are still to be fully explained [82].

## Nanoparticles penetration of the brain barriers and difficult to reach tissues or cells

Infections of the brain are often very difficult to treat because of the difficulty of most antimicrobial agents to cross the blood brain barrier and inhibit microbial agents [84]. This is due to the fact that the brain is made up of complex cell networks that filter foreign materials, protect and prevent the brain from injuries and diseases [83]. However, some small microbes such as viruses as well as some bacteria are still capable of bypassing and crossing the blood brain barrier [83, 84]. Substances entering the brain are mediated through a tight regulated systematic process of membrane transporters [82–84]. This tight regulatory system prevents most pharmacological antimicrobial agents from crossing the blood brain barrier and exercising their pharmacological activities [82–84]. In this regard nanotechnological antimicrobial agents could bring a novel dimensional approach that is capable of overcoming and bypassing the complex brain cell network, and inhibiting the brain pathogens, thus reducing the burden of microbial brain infections [85]. The NPs can potentially carry and potentially deliver antimicrobial across the blood brain barrier. In fact, it is known that NPs have very small nanosizes that exhibit vast physiochemical multifunctional properties that play a significant role of crossing the blood brain barrier with ease. These features of being able to transiting difficult biological system with ease without disrupting or damaging the cell membranes and sustaining the antimicrobials have made NPs and/or nanomaterials (nano-functionalized-ligands) very attractive for biomedical applications [5, 86]. For example, a single oral administration of poly-lactide-co-glycolide NP-encapsulated antituberculosis drugs consisting of rifampicin + isoniazid + pyrazinamide + ethambutol conjugate in murine mice was found to cross the blood brain barrier and sustained for 9 days in the brain [86]. Furthermore, based on colony forming unit enumerations and pathological examinations, the study showed that 5 oral doses administered every 10th day improved the pharmacologic activities of the polymer NP-antituberculosis drugs resulting in an undetectable level of *Mycobacterium tuberculosis* in the mice meninges [86].

The mechanisms of action of how the polymer- antituberculosis nanomaterials bypassed the complex cell network of blood brain barriers are yet to be uncovered. It is envisaged that the development of emerging novel NP-antimicrobial agents will soon revolutionize clinical medicine [86]. It is anticipated that the crossing of the blood brain barrier by NP-antimicrobial agents including other classes of drugs would reduce the burden of infections including meningitis caused by vast majority of pathogens.

## Nanoparticles enhancement of antimicrobial activities of other agents

The NPs play a significant role in enhancing the activities of other agents leading to effective and efficient treatment action. For example, the combination of SWCNTs and hydrogen peroxide (H_2_O_2_) or NaOCl increases the sporicidal effect on the spores of organisms such as *Bacillus* species when compared to treatment with H_2_O_2_ or NaOCl alone at the same concentrations [87]. In such treatments, synergistic mechanisms of efficacy are established due to contribution of multiple antimicrobial effects. Further analysis shows that SWCNTs do not only play the role of antimicrobial effect, but also increases permeability/susceptibility of the *Bacillus* species pathogen to H_2_O_2_ or NaOCl, thus significantly developing high effective sporicidal effect [87]. Furthermore, findings by Gilbertson et al. [6] found that oxygen functional groups when functionalized on MWCNTs, enhances several MWCNT properties such as redox activity, electrochemical and antimicrobial activities. The redox activities include the ability to enhance the oxidation of glutathione, and the reduction of surface carboxyl groups that promote the functional performance of MWCNTs antimicrobial activities for biomedical application [6]. This synergetic effect has equally been shown by AgNPs which enhanced the angiogenic properties of natural latex rubber for cell growth and wound healing [51].

## Nanoparticles disinfectants

The inventive approach of nanomaterials as disinfectant relate to their stability, homogeneity, high efficiency and efficacy of broad biocide spectrum of virucidal, bactericidal, fungicidal, antiparasitic and sporicidal as well as mycobactericidal and mycoplasmicidal potentials [88–90]. These excellent disinfectant properties as well as the additional ability of NPs surface functionalization and the dispersion on the NPs surfaces have been exhibited by a wide range of NPs [5–7]. Such functional groups provide very potent additional antimicrobial properties and include ligands such as hydroxyl, carboxyl, amine, and other chemical radicals [5]. The NPs including those of silver, copper and gold [91] have excellent cleaning and disinfecting properties. Some of these NPs are now being used as cleaning disinfectants in hospitals. In such instances, the surfaces may be coated with potent nanomaterials against nosocomial pathogens including the stubborn multi-drug resistant pathogens of Methicillin-resistant *Staphylococcus aureus (*MRSA) that are responsible for most nosocomial infections [88, 92]. For example, silicone polymers of AuNPs have shown to actively reduce the microbial load on clinical surfaces, particularly, when the surfaces are activated with white light [93].

To minimize the risk of microbial and other contamination of hospital HCW during various clinical procedures and examination procedures, hospital protective equipment are re-enforced with nanomaterials-antimicrobial agents that have been developed. Some of the HCW antimicrobial protective materials include surgical mask, gloves and many other latex personal protective equipment (PPE). For example, mixtures of silver nitrate and titanium dioxide NP coated on hospital facemask used during very delicate clinical procedures have shown to have significant protection against infectious agents [91, 93, 94]. The use of NPs-antiseptics has also led to an increase in surface area to volume ratio, thus improving the lethal action of NPs-antiseptics against pathogens [91, 93].

As a result of the biocidal action and non-toxic nature of some NPs such as AgNPs, they are widely coated on medical devices to reduce infections [95]. In addition, nanomaterials of silver are being used in pet-animal shampoos as disinfection, cleaning and softening agents [96]. The AgNPs can also be coated on filters used for the purification of water. In some studies, PVK and SWNTs were found to destroy bacterial cell membranes [34].

Furthermore, NPs are currently being used as preservatives in packages to prevent food spoilage. For example, allyl isothiocyanate (AIT) and CNTs can be incorporated into packaging materials so as to prevent the contamination of food by *Salmonella choleraesuis* [97]. The allyl isothiocyanate (AIT) and CNTs work by providing an antimicrobial film that reduces the microbial contamination, control oxidation and reduces the colour changes for up to 40 days [97].

## Nanoparticles antimicrobial mechanisms of action

Traditionally, most antimicrobial agents inhibit microbial growth through several mechanisms such as cell wall inhibition and lysis, inhibition of protein synthesis, alteration of cell membranes, inhibition of nucleic acid (NA) synthesis and antimetabolite activity [113]. The NP-antimicrobials, on the other hand, may encompass and differ slightly due to their vast physiochemical properties with respect to size, shape, surface area, surface energy, charge, crystallinity, agglomeration, aggregation and chemical composition [114–116]. Although most NP-antimicrobial mechanisms of action are still unknown and are currently under investigations [117], studies show that NPs can mediate bacterial cell membranes degradation [118–120]. For example, Li et al. [120] found the degradation of *S. aureus* by Catechin-Cu NPs. The Catechin-Cu NPs was also found to exert different mechanisms of action during *E. coli* cell wall degradation, which is an indication of different impacts on the Gram negative and Gram positive bacteria [120]. The multiple effects have also been observed in CuNP-antimicrobial actions which include the generation of reactive oxygen species and lipid peroxidation [118]. Other CuNP-antibacterial actions include protein oxidation and DNA degradation in *E. coli* cells [118]. Another study by Xie et al. [121] showed that zinc oxide (ZnO) NPs exerted bactericidal effect by disruption of the cell membrane and oxidative stress in *Campylobacter jejuni*. The NP-antimicrobials such as AgNP have also been shown to bind to lippopolysaccharides, surface proteins or porin, collapsing the microbial cell wall and limiting the membrane potential [122]. Similarly, AgNP have been found to induce efflux of phosphate, reduction of cellular ATP level, interacting with sulphahydryl (or thiol) group and altering cytoplasmic components as well as inhibiting the respiratory enzymes and blocking of DNA replication in both Gram negative and Gram positive bacterial pathogens [122]. These studies show that different NPs have very different physiochemical properties and thus exhibit different antimicrobial mechanisms of action.

## Nanoparticles toxicity

The NPs antimicrobial agents have excellent potent and low tendency of inducing resistance when compared to non-NPs-antimicrobial agents [123]. However, the NP-antimicrobial agents’ pharmacological properties may be hampered by potential toxicity [123, 124]. As stated in previous sections of this review paper, NPs facilitate the penetration and delivery of antimicrobial agents into biological membranes including microbial cells, thereby enhancing and increasing biological activities [76, 113]. This means that the toxicity of different NP-antimicrobial polymers needs a time-dependent understanding and characterization [125]. Generally, antimicrobial agents’ biocompatibility inhibition cannot occur without producing some undesirable health effects, either local or systemic. In fact, the most deterring effect of most drugs is their potential toxicity to organisms of which NPs-antimicrobials agents are not an exception. Therefore, effective NP-antimicrobial agents’ dose-related response is an important factor in relation to human exposure and other organisms. Few studies have described the toxicity of NP-antimicrobials (Table 1) with controversies. For example, a study by Cooper and Spitzer [126] shows that AgNPs antimicrobials at sub-lethal dose disrupt cytoskeleton and neurite dynamics when cultured in adult neural stem cells. For example, at sub-lethal dose of 1.0 μg/mL, AgNP cultured in neural stem cells induced the formation of f-actin inclusions, indicating a disruption of actin function [126]. Similar findings were reported by Baram-Pinto [101] that AgNPs capped with Mercaptoethane Sulfonate showed some serious effects in mammalian cells. Some results showed that PVK-SWCNT-antimicrobial agents were nontoxic to fibroblast cells as opposed to pure SWCNTs [34]. Multivalent Sialic acid functionalized AuNPs-antimicrobials agents have also been shown to demonstrate no toxic effect on Madin-Darby canine kidney cells [107]. Similarly, copper (II) nanohybrid solids-antimicrobial have shown no toxic effect on human hepatocellular carcinoma cells [109]. In another study, no cytotoxicity was reported when rats were treated with antibacterial AgNP-loaded titanium nanotube [127]. The rat cells expressed no toxicity thus demonstrating the competence of NPs-antimicrobials as future antimicrobial agents. However, despite several studies, the current available information is insufficient to ascertain the adverse effects of NP-antimicrobials on human health. Therefore, it is imperative that further research is carried out to mitigate any toxicological problems that may arise.

## Summary and future perspectives

Research has shown that the functionalization-immobilization and/or hybridization of NPs can enhance and improve the antimicrobial activities of the nanomaterials against a wide range of multi-resistant strains of pathogenic microorganisms. For example, a single type of NP-antimicrobial agent could show multiple antimicrobial properties against many pathogens. However, these characteristics may also alter the microbial flora of the body since their antimicrobial action is non-specific. Most of the studies reviewed showed that AgNPs were the widely used and have several antibacterial, antiviral, antifungal, anti-parasite, anti-insect and anti-vector borne properties. Generally, most NP-antimicrobial drugs were able to target and transit difficult membrane barriers, deliver and sustain the NP-antimicrobial doses resulting in disease clearance which is a difficult phenomenon for conventional antimicrobials. However, more information on the toxicological effects of NP-antimicrobial agents is needed so as to enhance and broaden their biomedical application [76]. In some instances, depending on the size of the NP, the particle tended to be toxic rather than demonstrating antimicrobial effect of inhibiting pathogens. For example, very small AgNPs were found to cover the pathogen, inhibiting oxygen supply to the pathogen thus reducing respiration and toxically killing the pathogen rather than inhibiting the microbial growth [14]. However, very small NPs may also be toxic to human pathogens. For example, AgNPs ranging from 10–20 nm were found to be toxic to *Bacillus* specie*s, E. coli* and *Staphylococcus* species. [14]. Therefore, it is imperative that further research is carried out to mitigate such problems.
